# Markers of Inflammation, Tissue Damage, and Fibrosis in Individuals Diagnosed with Human Immunodeficiency Virus and Pneumonia: A Cohort Study

**DOI:** 10.3390/pathogens13010084

**Published:** 2024-01-18

**Authors:** Katherine Peña-Valencia, Will Riaño, Mariana Herrera-Diaz, Lucelly López, Diana Marín, Sandra Gonzalez, Olga Agudelo-García, Iván Arturo Rodríguez-Sabogal, Lázaro Vélez, Zulma Vanessa Rueda, Yoav Keynan

**Affiliations:** 1 Escuela de Microbiología, Universidad de Antioquia, Medellin 050010, Colombia; katherine.penav@udea.edu.co; 2 Grupo de Investigación en Salud Pública, Facultad de Medicina, Universidad Pontificia Bolivariana, Medellin 050010, Colombia; anderson.riano@udea.edu.co (W.R.); lucelly.lopez@upb.edu.co (L.L.); dianamarcela.marin@upb.edu.co (D.M.); zulma.rueda@umanitoba.ca (Z.V.R.); 3 Grupo Bacterias & Cáncer, School of Medicine, Universidad de Antioquia, Medellin 050010, Colombia; omaria.agudelo@udea.edu.co; 4 School of Medicine, Universidad de Antioquia, Medellin 050010, Colombia; ivan.rodriguezmd@gmail.com; 5 Department of Medical Microbiology and Infectious Disease, University of Manitoba, Winnipeg, MB R3E 0J9, Canada; mariana.herreradiaz@umanitoba.ca (M.H.-D.); sandragonzalez0722@gmail.com (S.G.); 6 School of Medicine, Universidad Pontificia Bolivariana, Medellin 050010, Colombia; 7 JC Wilt Infectious Diseases Research Center, Winnipeg, MB R3E 3L5, Canada; 8 Infectious Diseases Section, Hospital Universitario San Vicente Fundación, Medellin 050010, Colombia; lazaro.velez@udea.edu.co; 9 Grupo Investigador de Problemas en Enfermedades Infecciosas-GRIPE, Facultad de Medicina, Universidad de Antioquia, Medellin 050010, Colombia; 10 Department of Internal Medicine, University of Manitoba, Winnipeg, MB R3E 0J9, Canada; 11 Department of Community Health Sciences, University of Manitoba, Winnipeg, MB R3E 0J9, Canada

**Keywords:** inflammation, cytokines, pulmonary dysfunction, HIV, pneumonia

## Abstract

Previous studies have noted that persons living with human immunodeficiency virus (HIV) experience persistent lung dysfunction after an episode of community-acquired pneumonia (CAP), although the underlying mechanisms remain unclear. We hypothesized that inflammation during pneumonia triggers increased tissue damage and accelerated pulmonary fibrosis, resulting in a gradual loss of lung function. We carried out a prospective cohort study of people diagnosed with CAP and/or HIV between 2016 and 2018 in three clinical institutions in Medellín, Colombia. Clinical data, blood samples, and pulmonary function tests (PFTs) were collected at baseline. Forty-one patients were included, divided into two groups: HIV and CAP (n = 17) and HIV alone (n = 24). We compared the concentrations of 17 molecules and PFT values between the groups. Patients with HIV and pneumonia presented elevated levels of cytokines and chemokines (IL-6, IL-8, IL-18, IL-1RA, IL-10, IP-10, MCP-1, and MIP-1β) compared to those with only HIV. A marked pulmonary dysfunction was evidenced by significant reductions in FEF25, FEF25-75, and FEV1. The correlation between these immune mediators and lung function parameters supports the connection between pneumonia-associated inflammation and end organ lung dysfunction. A low CD4 cell count (<200 cells/μL) predicted inflammation and lung dysfunction. These results underscore the need for targeted clinical approaches to mitigate the adverse impacts of CAP on lung function in this population.

## 1. Introduction 

The HIV global burden remained significant by the end of 2022, with the number of infected people reaching 39 million, and 1.3 million new infections reported [1,2]. Moreover, 630,000 people died from AIDS-related illnesses during this year [2]. At the end of 2022, 29.8 million people were accessing antiretroviral therapy, accounting for 76% of all people living with HIV (PLHIV) receiving treatment [3], up from 7.7 million in 2010. In Colombia, the trend in the prevalence of PLHIV continues to rise, reaching 166,496 cases reported in 2023, of which 82.68% were receiving antiretroviral therapy (ART) [4].

Despite tremendous efforts and progress in managing HIV and preventing progression to immune deficiency with effective antiretroviral therapy, pulmonary infections continue to pose a serious threat for PLHIV, since they represent a factor associated with morbidity and mortality in these patients. Tuberculosis and bacterial pneumonia contribute to approximately 80% of the hospital admissions among PLHIV [5,6].

Pneumonia is common in PLHIV due to the immune alterations and immunosuppression induced by the HIV infection, and the diminished immune response to protective vaccines resulting in increased susceptibility to infection (opportunistic or not) [7].

Infections associated with HIV lead to the activation of the monocyte–macrophage system and initiate an early response characterized by the production of proinflammatory cytokines and chemokines such as interleukin (IL)-1, IL-6, IL-8, IL-15, tumour necrosis factor (TNF)-α, granulocyte–macrophage colony-stimulating factor (GM-CSF), and macrophage inflammatory protein (MIP)-lα, among others, and an increase in CD4+ T lymphocytes and interferon (IFN)-γ. The latter acts as a chemoattractant for more leukocytes, affecting their differentiation and regulation [8,9,10]. In acute pneumonia, the activation of neutrophils, which act as first responders, results in the release of autocrine cytokines and chemokines such as IL-8, IL-4, IL-6, IL-10, IL-1β, transforming growth factor (TGF)-β, and TNF-α, among others [11,12,13]. In both cases, if such a response is not regulated, excessive inflammation can cause irreparable damage [11,13]. Cytokine and chemokine concentrations pre-HIV infection correlate not only with the risk of HIV acquisition, but also with disease progression and end organ dysfunction [14].

In the present study, we sought to correlate markers of inflammation, tissue damage, and fibrosis, in individuals living with HIV with and without pneumonia, with their pulmonary lung dysfunction.

## 2. Materials and Methods

### 2.1. Study Design and Setting

Data and samples from patients that participated in a prospective cohort study conducted between September 2016 and December 2018 in three highly complex hospitals in Medellín, Colombia (Hospital San Vicente Fundación, Clínica SOMA, and Clínica Universitaria Bolivariana) were used for this study. The analyses performed for this manuscript are focused on the information and samples obtained at the time of hospital admission (within 72 h of admission).

### 2.2. Participants

The cohort study included 17 patients living with HIV and diagnosed with community-acquired pneumonia (CAP), and 24 patients living with HIV without CAP.

### 2.3. Eligibility Criteria

#### 2.3.1. Inclusion Criteria

The inclusion criteria encompassed individuals ≥ 18 years old, living with HIV, and with or without CAP, who agreed to participate in the study and signed the informed consent form.

#### 2.3.2. Exclusion Criteria

In the group of patients with CAP, we excluded individuals with healthcare or ventilator-associated pneumonia, hospitalization in the last two weeks, antibiotics usage for more than 72 h in the previous week, causes of known immunosuppression other than HIV (use of prednisone ≥ 0.3 mg/kg/day for 3 weeks or more, or ≥1 mg /kg/day for more than seven days, or its equivalent in other steroids, cytostatic (except low-dose methotrexate: <15 mg/week), the presence of hematological neoplasms, granulocytopenia < 500 cells/mm^3^, obstructive pneumonia due to cancer, significant chronic lung disease (cystic fibrosis, severe chronic obstructive pulmonary disease (COPD) (FEV1 < 50%), bronchiectasis, or asthma), orotracheal intubation at study entry, major contraindication to obtaining induced sputum, the inability to perform spirometry due to a clinical condition, or those with a high probability of loss to follow-up [15].

### 2.4. Definitions

#### 2.4.1. Community-Acquired Pneumonia (CAP)

CAP was defined as acute lower respiratory tract infection associated with radiographic changes that were not explained by another condition, acquired outside the hospital.

#### 2.4.2. Human Immunodeficiency Virus (HIV) Infection

Having a HIV infection was defined as individuals with a positive serological demonstration using a fourth-generation HIV test (ELISA) (Siemens Healthcare Diagnostics, Aschaffenburg, Germany).

#### 2.4.3. Pulmonary Dysfunction (Outcome)

Pulmonary dysfunction was defined as a decrease in lung volume above reference values, according to the American Thoracic Society criteria, measured in terms of forced vital capacity (FVC)% predicted, forced expiratory volume in one second (FEV1)% predicted, and their ratio FEV1/FVC. The normal value for FEV1% predicted and FVC% predicted were defined as >0.8, and the normal value for FEV1/FVC was >0.7. Lung diseases groups are defined by spirometry patterns of FEV1% predicted, FVC% predicted, and FEV1/FVC values [16,17].

#### 2.4.4. FEF25 (Forced Expiratory Flow at 25% of the Forced Vital Capacity)

FEF25 was defined as the rate of airflow during the early phase of forced expiration, specifically measured at 25% of the forced vital capacity (FVC).

#### 2.4.5. FEF25-75 (Forced Expiratory Flow between 25% and 75% of the Forced Vital Capacity)

FEF25-75 was defined as the average rate of airflow during the middle phase of forced expiration, calculated between the 25% and 75% points of the forced vital capacity (FVC).

#### 2.4.6. FEV1 (Forced Expiratory Volume in 1 s)

FEV1 was defined as the volume of air forcefully exhaled during the first second of a forced expiratory maneuver, often used as a key indicator of lung function.

### 2.5. Procedures

#### 2.5.1. Admission

Patients were screened for eligibility and recruitment by physicians and a nurse at the participating hospitals during the study period. After informed consent was obtained, a baseline blood sample was collected, spirometry was performed, and clinical and sociodemographic information was collected.

#### 2.5.2. Blood Sample Collection

The samples were collected in sodium heparin tubes; plasma was separated and stored at −80 °C until processing in Winnipeg, Canada. 

#### 2.5.3. Spirometry

Pulmonary function values were obtained from the patients using a portable spirometer. The procedure followed the American Thoracic Society (ATS) guidelines [18]. A portable spirometer was used to measure forced vital capacity (FVC), FEV1, and the FEV1/FVC ratio. FEV1 is the maximum volume of air that can be forcefully exhaled in 1 s [19,20,21]. FVC is the total volume of air that can be forcefully exhaled. The FEV1/FVC ratio is the portion of a person’s vital capacity that is exhaled in the first second of expiration [19,20,21]. Each subject had to perform three successful attempts, but no more than eight consecutive maneuvers. FEV1% predicted and FVC % predicted were calculated using the actual measured values of FEV1 and FVC and their estimated FEV1 predicted and FVC predicted values. Predicted FEV1 and predicted FVC are estimated values from the general healthy population, based on gender, age, and height. Predicted FEV1% and predicted FVC% is a method of standardizing measured FEV1 and FVC by comparing them with estimated values from the general population [22].

#### 2.5.4. Data Collection

The following information was collected for all individuals: sex, age, occupation, origin, socioeconomic status and social security, comorbidities (COPD, diabetes, kidney injury, heart failure, liver disease, collagen disease, neoplasms, pregnancy, hypo and hypersplenism, epilepsy, impaired consciousness or swallowing, and previous pneumonia), clinical characteristics (respiratory symptoms, vital signs, weight, height, and presence of lymphadenopathies or organomegaly), vaccination status, prior use of antibiotics, smoking, consumption of psychoactive substances or alcohol, and radiological studies and laboratory tests (CD4 count, HIV viral load, blood chemistry, kidney and liver function, blood gases, and diagnostic tests for other sexually transmitted diseases such as syphilis and hepatitis B and C).

#### 2.5.5. Cytokines and Chemokines Selection and Detection

The cytokines/chemokines quantified in the study were selected based on previous results published in a systematic review by Head et al. in 2019 [23] and a pilot study published by Mao et al. in 2020 [24]. Three commercial assays were used to evaluate 17 immune markers: the Human Cyto/Chem/GF Panel, a kit used to detect gamma interferon-induced protein 10 (IP10), monocyte chemoattractant protein-1 (MCP1), macrophage inflammatory protein 1 alpha (MIP-1α), macrophage inflammatory protein 1 beta (MIP-1β), eosinophil chemotactic protein or eotaxin (ETXN), interleukin receptor antagonist -1 (IL-1RA), interleukin 1 beta (IL-1β), interleukin 6 (IL-6), interleukin 8 (IL-8), interleukin 10 (IL-10), interleukin 13 (IL-13), interleukin 17A (IL-17A), interleukin 18 (IL-18), vascular endothelial growth factor A (VEGFA); the Human Cyto/Chem/GF Panel A, to detect chemokine ligand 5 (CCL5) or regulated on activation, normal T cell expressed and secreted (RANTES); and Neurodegenerative MAG Panel 3 to detect plasminogen activator inhibitor-1 (TPAI1) and the surface molecule that acts as a receptor sCD14 (soluble cluster of differentiation 14). The assays were performed according to the manufacturer’s instructions, using 50 μL per sample, and performed in duplicate. Standards were reconstituted and serially diluted to generate standard curves. Two controls with low and high concentrations, provided by the commercial kit, were included in each assay, and considered positive controls for the experiment. Results were analyzed in the BioPlex-200 instrument (Bio-Rad, Mississauga, ON, Canada), reported as mean fluorescence intensity, and converted to pg/mL or ng/mL using the BioPlex^®^ Manager version 6.0 (Bio-Rad, Mississauga, ON, USA).

### 2.6. Statistical Analysis

The data collected were analyzed using IBM SPSSª version 29 (SPSS Inc., Chicago, IL, USA) and Prism version 9.5.0. We used descriptive statistics (median (interquartile range, IQR) and n (%)) to report the variables of interest (cytokines/chemokines and clinical and sociodemographic variables), and the Mann–Whitney U test and student’s T-test were used to evaluate differences between the group with HIV and CAP, and the group with only HIV. Dot plots for cytokine molecules by groups were generated. Correlations between cytokine molecules and lung function measurements were evaluated using Spearman’s rank and Pearson correlation coefficients to assess the relationship between inflammation and lung dysfunction. The correlation was considered statistically significant if the *p*-value < 0.05.

## 3. Results

**Figure 1:**
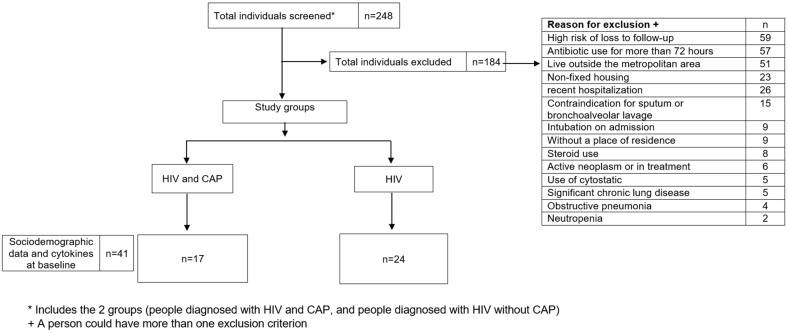
Flow diagram of the process of selection and exclusion of participants in the study.

A total of 248 patients were screened, and 41 patients were included in the cohort: 17 patients living with HIV and diagnosed with CAP and 24 patients who were living with HIV without CAP (Figure 1). However, 184 of these individuals were excluded due to various reasons, including a high risk of loss to follow-up, due to factors such as residence outside the metropolitan area, prolonged use of antibiotics, and contraindications for medical tests included in the study, as well as coexisting medical conditions, as specified in exclusion criteria. The reasons for exclusion are listed in Figure 1.

Table 1 shows the sociodemographic and clinical variables of patients living with HIV and diagnosed with CAP and patients who were living with HIV without CAP. Among the participants, 80.4% were men and 19.5% were women, with no significant characteristic differences between the groups (*p*-value= 0.178). The median age for the people living with HIV (PLHIV) with CAP was 36.50 (IQR 27.25–45.50) years, and 40.00 (IQR 32.50–53.00) for those without CAP. The ages of participants in the two groups ranged between 25 and 64 years, and the age distribution was similar (*p*-value= 0.943). The HIV and CAP group had a significantly higher proportion of patients with a low body mass index (BMI) (18.5 kg/m^2^) compared to the HIV group (62.5% vs. 8.3%, *p*-value= 0.001).

Most patients in both groups had taken antibiotics in the last 3 months; no significant difference was evident between the groups (*p*-value= 0.168). PLHIV without CAP reported a higher consumption of inhaled drugs (8.3%), smoked drugs (20.8%), antibiotics (100%), and cigarettes used in the last month (37.5%). The HIV and CAP group reported lower alcohol consumption in the last month than the HIV group (35.3% vs. 54.2%), although this difference was not statistically significant (*p*-value= 0.233). There were no significant differences in the rates of diabetes, chronic kidney injury, or congestive heart failure between the groups.

In addition, the HIV and CAP group had a significantly higher proportion of patients with a CD4 count less than <200 cells/µL, compared to the HIV group (66.7% vs. 30.4%, *p*-value < 0.003). No significant differences were found in the rates of high viral load between the two groups. In the HIV group, mycobacteria had lower predominance (12.5%) during admission, compared to the HIV and CAP group, where bacterial (33.3%) and mycobacterial (46.7%) infections predominated. No significant differences were observed between the two groups in terms of microbiological diagnosis, except for higher rates of tuberculosis among the group with HIV and CAP.

**Figure 2:**
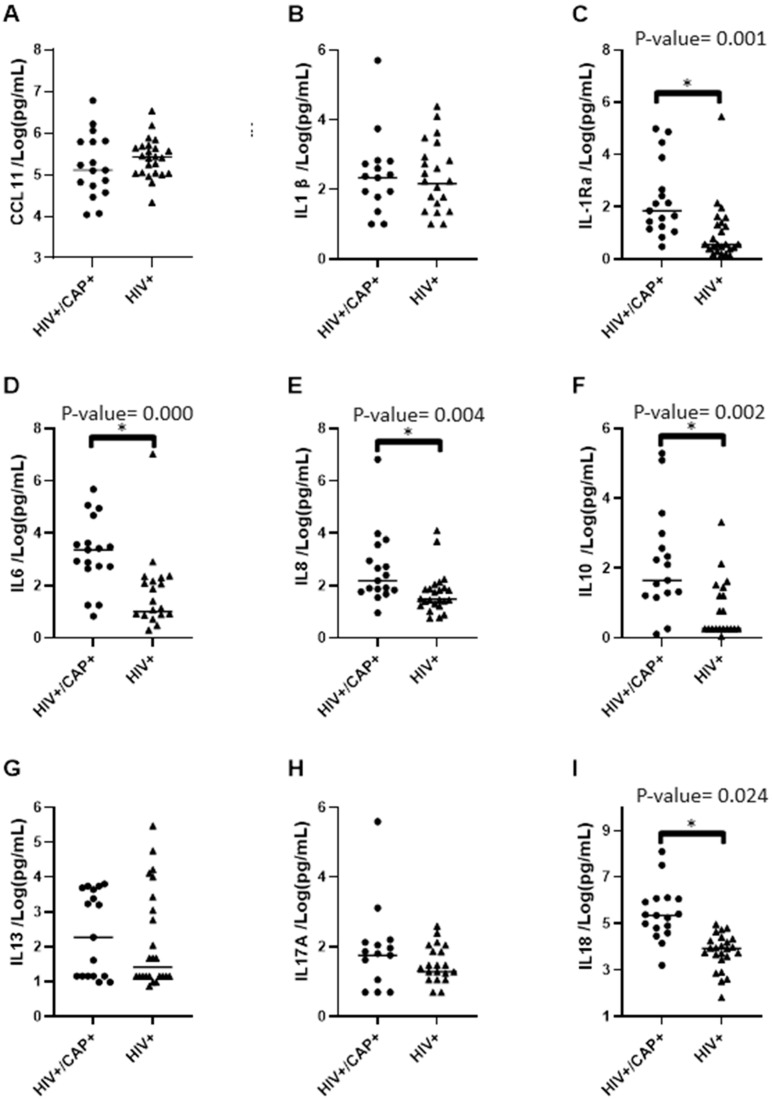

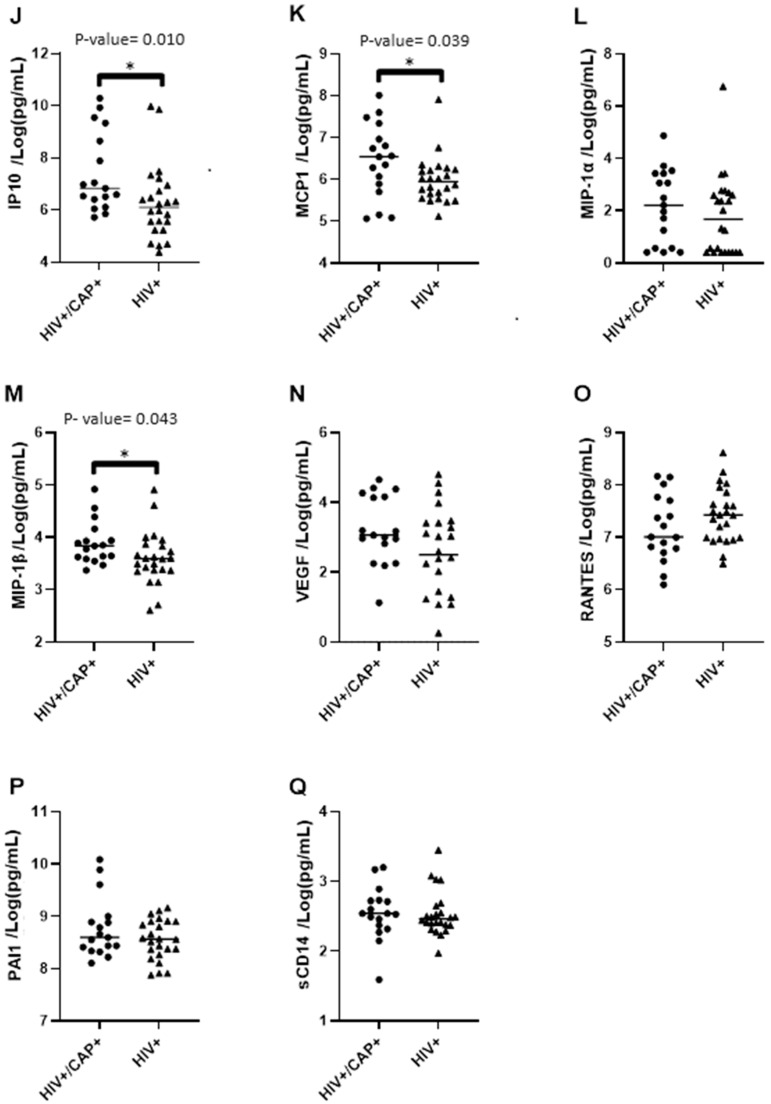
Plasma concentrations (natural logarithm) of (**A**) CCL11/eotaxin; (**B**) IL-1β; (**C**) IL-1Ra/IL1; (**D**) IL-6; (**E**) IL-8; (**F**) IL-10; (**G**) IL-13; (**H**) IL-17A; (**I**) IL-18; (**J**) IP10/CXCL10; (**K**) MCP1/CCL2; (**L**) MIP-1α/CCL3; (**M**) MIP-1β/CCL4; (**N**) VEGF/VPF; (**O**) RANTES/CCL5; (**P**) PAI1; and (**Q**) sCD14 measured in HIV and CAP compared to those with HIV but no pneumonia. Immunoassays were performed on plasma samples from all 41 participants included in the study at baseline. The vertical axis indicates the measured plasma concentration of each molecule in pg/mL. Each symbol represents a data point from a single individual (a circle indicates individual data points from the HIV and CAP group; a triangle indicates individual data points from the HIV group). At the time of admission, the concentrations of IL-6, IL-8, IL-10, IL-18, IL-1Ra/IL1, IP10/CXCL10, MCP1/CCL2, and MIP-1β/CCL4 were significantly higher in the HIV and CAP group than in the HIV group. The concentrations of the remaining molecules were not statistically different between the two groups at the time of the baseline. * Statistically significant if *p*-value < 0.05.

Differences in the proinflammatory response profile were determined by the plasmatic concentration of 17 cytokines and chemokines in HIV and CAP patients, and the HIV without pneumonia patients (HIV) (Supplementary Table S1). Firstly, it was observed that the concentrations of IL-1RA, IL-6, IL-8, IL-10, IL-18, IP10, MCP1, and MIP-1β were significantly higher in the HIV and CAP group than in the HIV group (*p*-value < 0.05) (See Supplementary Table S1 for details) (Figure 2). The concentrations of IL-13, IL-17A, MIP-1α /CCL3, PAI1, sCD14, IL-1β, CCL11/eotaxin, VEGF/VPF, and RANTES/CCL5 did not show significant differences between the groups. These findings suggest that an increased proinflammatory response with the alteration of particular cytokines and chemokines is established in people diagnosed with HIV, in the context of pneumonia.

**Figure 3:**
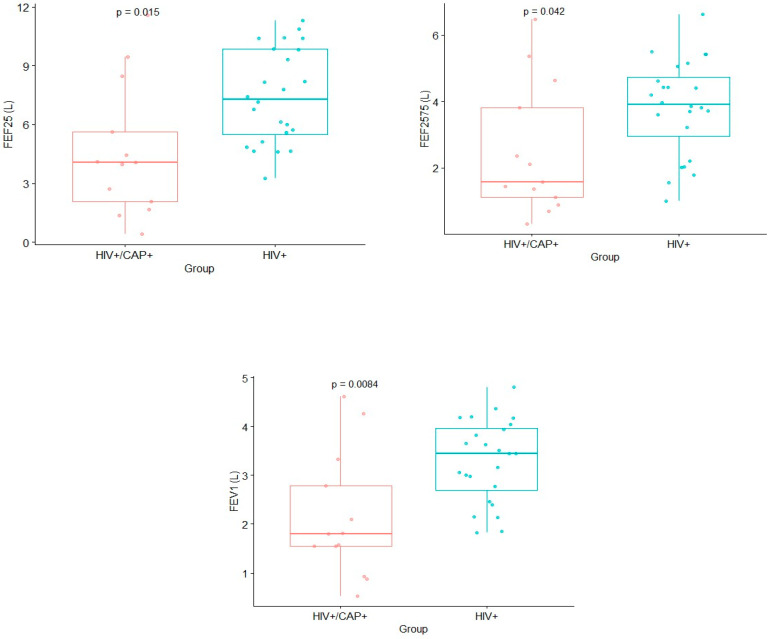
Lung function measures showing differences between groups.

The pulmonary function was explored by measuring FEF25, FEF25-75, FEV1, and FEV1/FVC in both groups of patients. FEF25, FEF25-75, and FEV1 were significantly lower in the HIV and CAP group compared to the HIV group, with *p*-values < 0.05 (Supplementary Table S2) (Figure 3). Nonetheless, no difference was observed for the FEV1/FVC measurement (Supplementary Table S2). These findings indicate that people with HIV and CAP exhibited a reduced pulmonary function compared to those with HIV only.

We further performed a correlation analysis between the parameters of lung function and the cytokines and chemokines evaluated in the HIV and CAP group and the HIV group (Supplementary Tables S3–S5). Supplementary Table S3 shows the results of the correlation coefficients between various cytokines and chemokines measured in plasma with non-normal distribution and the lung function variables FEF25, FEF25-75, FEV1/FVC, and FEV1 at baseline in the HIV and CAP group.

A negative correlation was found between IL-17A and FEV1 at baseline, with a correlation coefficient of −0.544 and a *p*-value of 0.055, suggesting that higher levels of IL-17A are associated with a reduction in lung function, as measured by FEV1 (see Supplementary Table S3 for details). On the contrary, cytokine IL-17A did not correlate with the variables FEF25, FEF25-75, and FEV1/FVC. Finally, no significant correlations were found between the lung variables (FEF25, FEF25-75, and FEV1/FVC) and the cytokines and chemokines (IL-6, IL-18, IL-10, IL-13, IL-1Ra/IL1, IP10/CXCL10, MCP1/CCL2, MIP-1α/CCL3, MIP-1β/CCL4, PAI1 and sCD14) in the HIV and CAP group.

Supplementary Table S4 presents the correlations between different biomarkers measured in plasma with normal distribution and the lung function variables FEF25, FEF25-75, FEV1/FVC, and FEV1 at the beginning of the study in the HIV and CAP group. A significant positive correlation of IL-1β with the FEV1/FVC ratio was observed, with a correlation coefficient of 0.615 and a *p*-value of 0.025. This suggests that, as plasma IL-1β levels increase, the lung function variable FEV1/FVC also tends to increase. Furthermore, a significant positive correlation of VEGF/VPF with FEV1/FVC ratio at baseline was observed, with a correlation coefficient of 0.593 and a *p*-value of 0.033. This indicates that, as plasma VEGF/VPF levels increase, the lung function variable FEV1/FVC also increases. The increase in VEGF was associated with a trend towards a lower FVC among the group with HIV and CAP; this might represent a more restrictive function. However, the trend did not reach significance and other parameters of interstitial/restrictive processes, such as the test of the diffusing capacity of the lungs for carbon monoxide (DLCO), were not captured in the study.

Although no significant correlations were found between IL-18, CCL11/eotaxin, and RANTES/CCL5, a positive trend was observed in the case of RANTES/CCL5.

Correlations between several biomarkers measured in plasma and multiple variables related to lung function in the HIV group are presented in Supplementary Table S5. There is a strong negative and statistically significant correlation between plasma IL-10 levels and the variables FEF25, FEF25-75, and FEV1 (with the correlation coefficient ranging between −0.428 and −0.710 and *p*-values of <0.05). Also, significant correlations were observed between the cytokine IP10/CXCL10 and several lung function parameters. The results indicate that, as IP10/CXCL10 levels increase, there is a trend towards a decrease in lung function. This is reflected in a reduction in forced expiratory flow to 25% (FEF25), 25–75% (FEF25-75), and in forced expiratory volume in 1 s (FEV1). These findings suggest that IP10/CXCL10 could serve as an important indicator of lung health in HIV patients and highlight the relevance of monitoring this cytokine in the context of lung function. Another marker associated with lung function was sCD14, demonstrating a significant negative correlation (*p*-value = 0.012) with FEV1/FVC, indicating that higher levels of sCD14 are associated with decreased FEV1/FVC, suggesting an adverse impact on overall lung function. Furthermore, a statistically significant negative correlation (*p*-value = 0.054) was found between sCD14 and FEV1, indicating that, as sCD14 levels increase, FEV1 tends to decrease, signaling a negative effect on FEV1-specific lung function. We also examined the relationship between FVC and the immune mediators and found that IL-6, IL-8, IL-10, IL-17A, IL-1Ra, and IP10 were all negatively correlated with the FVC (Supplementary Table S6). The interpretation of this result in itself is insufficient to comment about the restrictive pattern of lung function, due to the lack of measurement of total lung capacity or DLCO.

Lastly, the significant negative relationship (*p*-value 0.05) between sCD14 and FEF25-75 suggests that, as sCD14 levels increase, FEF25-75 tends to decrease, indicating a relationship between sCD14 and lung function in the range of FEF25-75, crucial for lung ventilation. Finally, no relationship is observed between the cytokines and chemokines (IL-6, IL-8, IL-13, IL-17A, IL-1Ra/IL1, MCP1/CCL2, MIP-1α/CCL3, MIP-1β/CCL4, and PAI1) and the parameters of lung function in the HIV group.

## 4. Discussion

The complex interaction between human immunodeficiency virus (HIV), pneumonia, and immunological processes has been an area of increasing interest in medical research. In this cohort study, we set out to correlate markers of inflammation, tissue damage, and fibrosis, in people living with HIV with and without pneumonia, with lung dysfunction.

This study yielded several key findings. First, patients with HIV and pneumonia exhibited elevated plasma levels of various cytokines, including IL-6, IL-8, IL-18, IL-1RA, IL-10, IP-10, MCP-1, and MIP-1β, compared to patients with HIV alone. Secondly, marked pulmonary dysfunction was evident in those with HIV and pneumonia, reflected in parameters such as FEF25, FEF25-75, and FEV1, indicating a reduction in airflow and lung capacity. Third, the relationship between the concentrations of nine specific cytokines (IL-6, IL-17A, MCP-1, PAI1, IL-1β, VEGF, IL-10, IP-10, and sCD14) and lung function parameters supports the hypothesis that pneumonia-associated inflammation could contribute to tissue damage and accelerated pulmonary fibrosis in patients with HIV. Furthermore, the identification of low CD4 values (<200 cells/μL) as an added risk factor suggests a greater susceptibility to infection and as previously reported, heightened inflammation and tissue damage. sCD14, a marker of monocyte activation, has been previously shown to reflect persistent immune activation and correlated with the occurrence of cardio-pulmonary complications [23]. We found that higher levels of sCD14 are associated with decreased FEV1/FVC, supporting the role of inflammation in airflow limitation. These results highlight the need for specific clinical and therapeutic approaches to mitigate the adverse impact on lung function in this population.

The inflammatory response plays a crucial role in the pathogenesis of pneumonia, especially in HIV-infected individuals, where immune function may be compromised [25,26,27]. In our study, patients with HIV and pneumonia exhibit immune dysregulation, characterized by elevated levels of proinflammatory cytokines (IL-6, IL-8, IL-18), anti-inflammatory cytokines (IL-1RA, IL-10), and chemokines (IP10, MCP-1, MIP-1β) in their plasma, compared to the HIV group.

Overall, these data indicate that HIV-associated pneumonia is accompanied by a high degree of immune activation [28]. This is consistent with previous studies reporting that various cytokines and chemokines, such as IL-6, IL-8, IL-10 and IP10, are elevated in patients with HIV and pneumonia [23,24,29,30]. These variations between groups could be due to several of the following reasons: (1) an acute and intense inflammatory response; (2) complications and severity of respiratory infection; (3) the interaction between HIV and pneumonia; (4) systemic inflammation and pulmonary response; (5) the modulation of the immune response; (6) the regulation of lung inflammation; (7) immune activation; (8) the antiviral and antimicrobial response; (9) neutrophil infiltration in the lungs; or (10) more advanced immune suppression due to advanced HIV in the group with HIV and CAP.

IL-6 (interleukin-6) is a proinflammatory cytokine that promotes inflammation and regulates the immune response [31,32,33]. Increased IL-6 levels have been observed in inflammatory diseases, and HIV infection has even been shown to induce the expression and secretion of IL-6 [23,32,34]. In our study, we observed a significant increase in IL-6 levels in patients with HIV, and this elevation is even more pronounced in those who have coinfection with pneumonia. This result suggests that the presence of pneumonia contributes significantly to the increase in IL-6 levels in patients with HIV, indicating an association between lung infection and a more pronounced inflammatory response that is potentially driven by lung infection. Different studies have reported that elevated levels of IL-6 have been associated with worse outcomes in community-acquired pneumonia [23,35,36,37]. IL-8 (interleukin-8) is a proinflammatory cytokine that plays a crucial role in leukocyte chemotaxis and in recruiting and activating neutrophils at sites of inflammation [38]. Elevated IL-8 levels have been associated with lung inflammation and pneumonia in HIV patients [23,39]. IL-10 (interleukin 10) is an anti-inflammatory cytokine that regulates the immune response and limits inflammation [40,41,42]. However, in some cases, elevated IL-10 levels may be associated with an inadequate immune response and an increased susceptibility to lung infections in HIV patients [35,40,43,44,45]. We found a significant negative correlation between IL-10 concentrations and FVC, a correlation that merits further investigation as it may point to a restrictive or preserved ratio lung dysfunction. A recent study identified that lower concentrations of IL-10 were associated with pulmonary impairment in a cohort of PLHIV from India [46]. IL-1Ra (interleukin 1 receptor antagonist) is a negative regulator of inflammation that acts by competing with interleukin-1 (IL-1) for its receptor and blocking its proinflammatory effects [47]. In HIV infection, the production of IL-1Ra has been shown to be induced in the early stages of infection [48]. Elevated levels of IL1-Ra may reflect an attempt to regulate the excessive inflammatory response in HIV-associated pneumonia [36,49,50]. IL-18 (interleukin-18) is a proinflammatory cytokine involved in the regulation of the immune response and inflammation [51,52,53]. Increased IL-18 levels have been observed in patients with HIV and pneumonia, suggesting an exacerbated inflammatory response [51,54,55]. IP10 (interferon gamma-inducible protein 10), also known as CXCL10, is a chemoattractive cytokine that attracts inflammatory cells, especially T lymphocytes, to the site of infection [56,57]. Increased IP10 levels have been associated with lung inflammation and an altered immune response in patients with HIV and pneumonia [23,27]. Our results agree with those reported by Wang et al. [30], who identified eight biomarkers, including markers of inflammation (soluble TNF receptor (sTNFR)-1, sTNFR-2, and C-reactive protein (CRP)), coagulation (D-dimer), the activation of T cells (sCD27), a response to interferon (IP-10), the activation of monocytes and macrophages (sCD14), and fibrosis (hyaluronan), and reported elevated levels of these among people coinfected with HIV and pneumonia.

Monocyte chemoattractant protein 1 (MCP-1), also known as CCL2, is a chemoattractant chemokine that recruits monocytes and dendritic cells to the site of infection [58,59]. Elevated levels of MCP-1 may indicate the excessive recruitment of inflammatory cells in the lungs of patients with HIV and pneumonia [50]. Sui et al. [60] reported high levels of MCP-1 in the lung tissue of macaques infected by the simian/human immunodeficiency virus (SHIV) with pneumonia and suggested that this chemokine plays a role in the recruitment of inflammatory macrophages in the lung. MIP-1β (macrophage inflammatory protein-1 beta), also known as CCL4, is a proinflammatory chemokine that plays a role in the attraction and activation of cells of the immune system, especially monocytes, lymphocytes, and natural killer cells [61]. There is little data on the role of this chemokine in patients coinfected with HIV and pneumonia. However, increased levels of MIP-1β have been associated with lung inflammation. Therefore, it could be related to the altered immune response observed in patients with HIV and pneumonia. Jambo et al. [50] have shown that bronchoalveolar lavage (BAL) fluid taken from treatment-naïve HIV-positive participants has higher concentrations of RANTES and TNF-β and a shift toward the MIP-1β, MCP-1, and IP10 signaling network [27,50]. On the other hand, Cocchi et al. [62] demonstrated that the mean levels of MIP-1α and MIP-1β released by CD8+ T cells are significantly lower among PLHIV and advanced disease, as defined by CD4+ T cell counts of less than 200 per μL or by clinical diagnosis, compared to asymptomatic PLHIV. Furthermore, the production of MIP-1α and MIP-1β by CD8+ T cells was correlated with CD4+ and CD8+ T cell counts. Like our findings, a considerable proportion of patients in both groups had CD4 < 200, indicating an advanced immunodeficiency state. Information on the distribution of CD4+ T cell counts in the HIV group and the HIV with pneumonia group is of great relevance, as it provides insights into the immune function of patients in each category. The higher proportion of individuals with CD4 > 500 among the group with HIV alone, compared to those with HIV and CAP, may underlie some of the observed differences, as advanced immune suppression among those diagnosed with pneumonia on its own is likely driving some of the heightened inflammation, whether driven by viral replication or the presence of an undiagnosed co-infection [63].

Accumulating evidence suggests that patients with an uncontrolled HIV infection, characterized by a CD4 count < 200 cells/mm^3^ and/or a detectable HIV viral load, face a significantly elevated risk of severe disease and mortality [64]. The elevated risk may be related to observed patterns of coinfections with mycobacterial, fungal, and bacterial agents, as well as significant comorbidities associated with advanced HIV disease. These additional conditions may modulate the immune response of patients, influence the levels of cytokines and chemokines, and contribute to the complexity of the responses observed in these cases. Concordant results were reported by Keynan et al. [29], where different inflammatory patterns were associated with the various categories of respiratory pathogens that caused pneumonia in people infected with HIV. Presumably, there are other factors that affect the expression of these cytokines in patients with HIV and pneumonia, which is the subject of future research.

We found lower BMIs among the group of individuals with HIV and CAP. The low BMI reflects advanced HIV disease, lower CD4 counts, and a potentially increased likelihood of coinfections. HIV infection has been associated with IL-6 and INFγ among underweight individuals [65]. It is not possible to separate the role of low BMI from overall poor health due to advanced HIV disease in this study.

Although the concentrations of IL-13, IL-17A, MIP-1α/CCL3, PAI1, sCD14, IL-1β, CCL11/eotaxin, VEGF/VPF, and RANTES/CCL5 did not exhibit significant differences between the HIV and pneumonia and HIV-only groups, several reasons could explain this lack of disparity. These molecules may be involved in specific and complex immune responses, influenced by factors such as the specific timing of measurements, inherent biological variability, complex interactions between cytokines, and the possible influence of other factors, such as comorbidities and medical treatments. Furthermore, their roles in different stages of HIV infection and pneumonia could contribute to the lack of differences observed in this study, highlighting the need for more detailed investigations and the consideration of multiple variables for a comprehensive understanding of these results.

The study documents, as expected, that participants diagnosed with HIV and pneumonia have a more impeded lung function than the HIV group. This is evidenced by the significant differences observed in measures such as FEF25, FEF25-75, and FEV1, which suggest an additional negative impact on lung function when co-infection with pneumonia occurs in individuals living with HIV. The deterioration of lung function after pneumonia is consistent with previous findings [66,67]. A work by North et al. [68] that investigated HIV infection, tuberculosis, and COPD in a Ugandan cohort found a higher prevalence of COPD in HIV-positive people with a history of tuberculosis. These findings are consistent with our results and suggest that the decline in lung function in HIV infection could be attributed to lung co-infection rather than HIV infection. A high proportion of both groups were smokers, but the distribution of smoking between the groups was similar and does not appear to explain the observed differences in lung function. However, it is crucial to recognize that smoking alone may not be the sole factor influencing lung function. The intricate interplay of HIV infection, co-infection with pneumonia, and smoking could collectively contribute to the decline in lung function observed in our study. These findings underscore the need for a comprehensive approach in assessing respiratory health in individuals with HIV, considering the multifactorial nature of pulmonary complications. Further investigations, incorporating detailed analyses of additional risk factors and longitudinal assessments, are warranted to elucidate the complex interactions influencing lung function in this population.

A decrease in FEF25 and FEF25-75 could indicate obstructions in the small airways of HIV patients with pneumonia. In contrast, a decrease in the FEV1 indicates global obstructive lung function. Importantly, these differences could have several potential causes, such as lung inflammation associated with community-acquired pneumonia, side effects of antiretroviral therapy for HIV, or HIV infection itself. For a full understanding of the clinical implications of these differences, additional analyses and the consideration of other clinical and patient demographic factors may be necessary, as is the assessment of response to bronchodilators, which can shed light on the reversibility of the obstruction. However, it is important to note that other measures did not show significant differences, highlighting the complexity of the relationship between HIV, pneumonia, and lung function. Morris et al. [69] observed that individuals living with HIV and with *Pneumocystis jirovecii* or bacterial pneumonia had a permanent decrease in FEV1, highlighting the need for longitudinal follow-ups.

Various studies indicate that people with HIV face a higher prevalence of lung disease due to viral replication, the maintenance of a chronic inflammatory state, and decreased immunity compared to individuals without HIV [70,71]. Research by Gingo et al. [66] revealed that parameters such as a forced expiratory volume in 1 s/forced vital capacity (FEV1/FVC) ratio less than 0.7 and a pulmonary diffusion capacity (DLCO) less than 60% were associated with an increase in mortality from various causes in people with HIV. Likewise, in the context of pneumonia, immune dysregulation and lung damage are observed. Upon the invasion of pathogens into the lungs, neutrophils are recruited as first responders, triggering the release of local and systemic cytokines, such as IL-4, IL-6, IL-10, IL-8, IL-1β, TNF-α, and TGF-β. This process, strongly influenced by the invading microorganism, can trigger uncontrolled inflammation that can lead to collateral damage resulting in tissue remodeling, fibrosis, and lung dysfunction [11,12,23].

In our study, we performed a correlation analysis in the group of patients with HIV and pneumonia, evaluating the cytokines IL-6, IL-17A, MCP1/CCL2, PAI1, IL-1β, and VEGF/VPF in relation to key parameters of lung function. IL-1β and VEGF/VPF were correlated with a high FEV1/FVC ratio. This result may be in keeping with the role VEGF plays in interstitial lung disease and the development of pulmonary fibrosis, as this ratio suggests a restrictive pattern [72]. We did not measure DLCO and did not obtain high-resolution chest computed tomography and we cannot make a definitive determination. Similarly, IL-1β was associated with a decline in lung volume in a Danish cohort [73] and IL-1β induced lung tissue damage, leading to fibrosis [74]. Furthermore, we extended this analysis to the group of HIV patients, examining the cytokines IL-10, IP10/CXCL10, and sCD14 in relation to various parameters of lung function. We also found significant correlations, suggesting a potential association between these cytokines and lung function in the context of HIV infection.

These findings underscore the importance of understanding and managing inflammatory responses in the context of lung function in patients with HIV and pneumonia. Targeting and modulating these inflammatory responses could be a key strategy for adjunctive therapy that can improve lung function in this population. Furthermore, the identification of these relationships provides a basis for additional research aimed at developing specific therapeutic approaches that can attenuate inflammation and preserve lung health in patients with HIV and respiratory complications.

This study has several limitations that should be considered when interpreting the results. First, the retrospective nature of the design limits the ability to establish causal relationships between cytokines and lung function. Furthermore, the heterogeneity of the population studied, the limited ability to identify the causative organism leading to pneumonia (a common feature of pneumonia studies) [75], and the inclusion of different clinical profiles and previous treatments, could introduce biases and make the generalization of the findings difficult. Cytokines were measured systemically, and, therefore, may not represent the processes that occur in the lung compartment, and they are affected by a myriad of preexisting or coexisting conditions and coinfections, making the understanding of cause and effect complicated. The lack of longitudinal data also limits the ability to assess changes over time. Furthermore, the absence of certain inflammatory markers in the analysis may not fully reflect the complexity of the immune system in relation to lung function in patients with HIV and pneumonia. The inherent difference between the study groups, with more advanced immune dysfunction among those diagnosed with HIV and CAP, influences the results of cytokines and spirometry; however, it does reflect the “real life” differences between groups.

Despite the limitations, this study provides valuable information on the correlation between cytokines and lung function in patients with HIV and pneumonia. The inclusion of detailed measures of lung function and a comprehensive analysis of multiple cytokines provides a comprehensive view of the interplay between the immune system and lung health in this clinical context. Furthermore, the identification of potential biomarkers associated with lung function in this specific group of patients may support risk stratification and could guide future research and therapeutic strategies focused on modulating inflammatory responses to improve lung health in individuals with HIV and pneumonia coinfection.

## 5. Conclusions

In conclusion, this study has shed light on the complex interrelationship between cytokines and lung function in patients with HIV and pneumonia coinfection. Although significant correlations were observed between various cytokines and lung function parameters, the inherent limitations of the observational design and the heterogeneity of the population studied emphasize the need to interpret these findings with caution. The influence of clinical factors and prior treatments underscores the importance of future longitudinal and prospective research to better understand dynamics over time.

## Figures and Tables

**Table 1:** Baseline sociodemographic and clinical characteristics of the 41 participants in the study, according to PLHIV and CAP group (n = 17), and PLHIV without CAP group (n = 24).

Groups						
Variable		HIV and CAP (n = 17)		HIV (n = 24)		*p*-Value
		n	%	n	%	
Sex	Female	5	29.4	3	12.5	0.178
	Male	12	70.6	21	87.5	
Age, years	<24.9	2	11.8	3	12.5	0.943
	25–64	15	88.2	21	87.5	
BMI (kg/m^2)^	18.5	10	62.5	1	8.3	0.001 *
	18.51–24.99	5	31.3	16	66.7	
	>24	1	6.3	6	25.0	
Taken antibiotics in the last 3 months		2/3	66.7	5/5	100.0	0.168
Inhaling drugs used in the last month		1/17	5.9	2/24	8.3	0.767
Smoking drugs used in the last month		2/17	11.8	5/24	20.8	0.447
Cigarettes used in the last month		4	23.5	9	37.5	0.344
Liquor consumption in the last month		6/17	35.3	13/24	54.2	0.233
Cigarettes/day	<5	15	88.2	17	70.8	0.319
	6–15	2	11.8	5	20.8	
	>16	0	0.0	2	8.3	
Diabetes		1	5.9	1	4.2	0.802
Chronic renal injury		0	0.0	2	8.3	0.222
Congestive cardiac failure		0	0.0	2	8.3	0.222
Microbiological diagnosis	Bacteria	5	33.3	0	0.0	0.0352 *
	*Mycobacterium tuberculosis*	7	46.7	3	12.5	
	Mycosis	3	20.0	1	25.0	
CD4 count < 200 (cells/µL)		10/15	66.7	7/23	30.4	0.003 *
Viral load > 100,000 (copies/µL)		6/16	37.5	6/16	37.5	1.000

* Statistically significant if *p*-value < 0.05.

## Data Availability

The data presented in this study are available on request from the corresponding author. The data are not publicly available due to ethical reasons, because, at the time people were included in the study, we did not request their permission to share their data publicly.

## Supplementary Materials

The following supporting information can be downloaded at: https://www.mdpi.com/article/10.3390/pathogens13010084/s1. Supplementary Table S1: concentration of cytokines/chemokines measured in the plasma samples of the HIV and CAP group (n = 17) and the HIV group (n = 7) at study baseline; Supplementary Table S2: indices for lung function test measured using portable spirometry for HIV and CAP group and HIV group at the time of hospital baseline; Supplementary Table S3: correlations between the concentration of cytokines/chemokines with non-normal distribution measured at baseline and lung function test indices (FEV1) in the HIV and CAP group; Supplementary Table S4: correlations between the concentration of cytokines/chemokines with normal distribution measured at baseline and lung function test indices (FEV1/FVC) in the HIV and CAP group; Supplementary Table S5: correlations between the concentration of cytokines/chemokines with non-normal distribution measured at baseline and lung function test indices (FEF25, FEF25-75, FEV1/FVC, and FEV1) in the HIV group; Supplementary Table S6: correlations between the cytokines/chemokines concentration and forced vital capacity (FVC).
