# Supplementary material for: Markers of Inflammation, Tissue Damage, and Fibrosis in Individuals Diagnosed with Human Immunodeficiency Virus and Pneumonia: A Cohort Study
Source: Pathogens. 2024 Jan 18;13(1):84. doi: 10.3390/pathogens13010084 (PMC10820350; doi:10.3390/pathogens13010084)
Supplement: Supplementary file 1 [file pathogens-13-00084-s001.zip › pathogens-2816721-supplementary.pdf]

Supplementary material

Table S1. Concentration of cytokines/chemokines measured in the plasma samples of the HIV and CAP group (n=17) and the HIV group (n=7) at study baseline.

	**Study groups**		
	**HIV and CAP**	**HIV**	
Cytokines/ Chemokines	***Baseline median (IQR)***		***p-value****
IL-6	3.371(2.683-4.155)	0.992(0.340-2.146)	0.000*
IL-8	2.193(1.795-3.258)	1.478(1.249-1.897)	0.004*
IL-10	1.647(1.184-2.777)	0.262(0.262-1.206)	0.002*
IL-13	2.270(1.163-3.665)	1.416(1.163-3.333)	0.695
IL-17A	1.746(0.691-2.088)	1.280(0.779-1.860)	0.569
IL-1Ra/IL1	1.850(1.192-3.270)	0.562(0.393-1.366)	0.001*
IP10/ CXCL10	6.832(6.257-8.993)	6.100(5.311-6.898)	0.010*
MCP1/ CCL2	6.537(5.795-7.147)	5.944(5.575-6.221)	0.039*
MIP-1α /CCL3	2.211(0.556-3.423)	1.676(0.405-2.664)	0.214
MIP-1β/CCL4	3.843(3.612-4.046)	3.597(3.373-3.861)	0.043*
PAI1	8.595(8.374-8.945)	8.561(8.297-8.901)	0.491
sCD14	2.543(2.344-2.723)	2.456(2.3747-2.621)	0.427
Cytokines/ Chemokines	***Baseline mean (SD)***		***p-value****
IL-1β[a]	2.123(1.405)	2.065(1.284)	0.223
IL-18[a]	5.427(1.175)	3.780(0.780)	0.024*
CCL11/eotaxin[a]	5.219 (0.766)	5.403(0.469)	0.485
VEGF/ VPF[a]	3.235(0.977)	2.418(1.431)	0.108
RANTES/CCL5[a]	7.172(0.632)	7.423(0.527)	0.154

Molecule concentration: pg/ml
Mann Whitney U test
Student t-test[a]
****Statistically significant if*** $p < 0.05$
HIV: human immunodeficiency virus
CAP: community acquired pneumonia

Table S2. Indices for lung Function test measured using portable spirometry for HIV and CAP group and HIV group at the time of hospital baseline.

Lung function variables	Groups		
	HIV and CAP group	HIV group	
	Baseline mean (SD)		p-value*
FEF25	4.609(3.360)	7.438(2.403)	0.015*
FEF2575	2.476(1.962)	3.823(1.439)	0.042*
FEV1/FVC	0.815(0.160)	0.842(0.063)	0.567
FEV1	2.132(1.269)	3.291(0.842)	0.008*

Spirometric variables expressed in liters.
Student t-test
*Statistically significant if $p < 0.05$
HIV: human immunodeficiency virus
CAP: community acquired pneumonia

Table S3. Correlations Between the Concentration of cytokines/chemokines with non-normal distribution measured at baseline with lung Function Test Indices (FEV1) in the HIV and CAP group).

	HIV and CAP Group							
	FEF25		FEF25-75		FEV1/FVC		FEV1	
Molecules measured in plasma	Correlation coefficient	P value	Correlation coefficient	P value	Correlation coefficient	P value	Correlation coefficient	P value
IL-6	-0.154	0.616	-0.071	0.817	-0.093	0.762	-0.198	0.517
IL-8	0.126	0.681	-0.110	0.721	0.203	0.505	-0236	0.437
IL-10	0.385	0.194	0.374	0.209	0.385	0.194	0.176	0.566
IL-13	-0.1	0.746	-0.290	0.336	0.396	0.181	-0.296	0.326
IL-17A	-0.291	0.334	-0.324	0.280	0.456	0.117	-0.544	**0.055***
IL-1Ra/IL1	-0.313	0.297	-0.324	0.280	0.077	0.803	-0.445	0.128
IP10/ CXCL10	-0.115	0.707	0.016	0.957	-0.467	0.108	-0.011	0.972
MCP1/ CCL2	-0.099	0.748	-0.143	0.642	-0.005	0.986	-0.203	0.505
MIP-1α /CCL3	-0.459	0.115	-0.381	0.199	0.061	0.844	-0.464	0.110
MIP-1β/CCL4	-0.368	0.216	-0.242	0.426	0.099	0.748	-0.291	0.334
PAI1	0.022	0.943	0.088	0.775	0.258	0.394	-0.038	0.901
sCD14	-0.385	0.194	-0.165	0.590	-0.214	0.482	-0.104	0.734

Molecule concentration: pg/ml
Spearman's Rho Correlation
*Statistically significant if $p < 0.05$
HIV: human immunodeficiency virus
CAP: community acquired pneumonia

Table S4. Correlations Between the Concentration of cytokines/chemokines with normal distribution measured at baseline with lung Function Test Indices (FEV1/FVC) in the HIV and CAP group.

	HIV and CAP Group							
	FEF25		FEF25-75		FEV1/FVC		FEV1	
Molecules measured in plasma	Correlation coefficient	P value	Correlation coefficient	P value	Correlation coefficient	P value	Correlation coefficient	P value
IL-1β	-0.152	0.620	-0.096	0.755	0.615	**0.025***	-0.293	0.331
IL-18	0.279	0.355	0.069	0.822	0.174	0.570	0.056	0.856
CCL11/eotaxin	-0.028	0.927	0.085	0.781	0.049	0.874	-0.053	0.863
VEGF/ VPF	0.139	0.650	0.241	0.428	0.593	**0.033***	0.098	0.749
RANTES/CCL5	0.268	0.376	0.366	0.218	0.522	0.067	0.243	0.425

Molecule concentration: pg/ml
Pearson's correlation coefficient
*Statistically significant if $p < 0.05$
HIV: human immunodeficiency virus
CAP: community acquired pneumonia

Table S5. Correlations between the concentration of cytokines/chemokines with non-normal distribution measured at baseline with lung Function Test Indices (FEF25, FEF25-75, FEV1/FVC and FEV1) in the HIV group.

HIV Group								
	FEF25		FEF25-75		FEV11/FVC		FEV1	
Molecules measured in plasma	Correlation coefficient	P value	Correlation coefficient	P value	Correlation coefficient	P value	Correlation coefficient	P value
IL6	-0.290	0.170	-0.270	0.201	-0.330	0.115	-0.335	0.110
IL8	-0.080	0.712	0.019	0.929	-0.045	0.834	-0.096	0.655
IL10	-0.710	**0.000***	-0.428	**0.037***	-0.218	0.307	-0.525	**0.008***
IL13	0.180	0.400	0.201	0.346	0.070	0.744	0.218	0.307
IL17A	0.067	0.755	0.154	0.473	0.122	0.571	0.048	0.822
IL-1Ra/IL1	-0.333	0.111	-0.293	0.164	-0.330	0.115	-0.260	0.219
IP10/ CXCL10	-0.422	**0.040***	-0.402	**0.052***	-0.357	0.087	-0.541	**0.006***
MCP1/ CCL2	-0.153	0.475	-0.054	0.802	0.038	0.859	-0.065	0.762
MIP-1α /CCL3	0.049	0.819	0.002	0.992	0.054	0.803	-0.049	0.819
MIP-1β/CCL4	0.028	0.897	-0.006	0.977	-0.086	0.689	0.182	0.394
PAI1	-0.003	0.990	-0.110	0.607	-0.019	0.929	-0.205	0.336
sCD14	-0.149	0.488	-0.398	**0.054***	-0.406	**0.049***	-0.506	**0.012***

Molecule concentration: pg/ml
Spearman's Rho Correlation
*Statistically significant if $p < 0.05$
HIV: human immunodeficiency virus
CAP: community acquired pneumonia

Table S6. Correlation between the cytokines/chemokines concentration and forced vital capacity (FVC).

	FVC	
Molecules measured in plasma	Correlation coefficient	P value
IL6	-0.471	**0.003***
IL8	-0.389	**0.017***
IL10	-0.524	**0.001***
IL13	-0.003	0.984
IL17A	-0.315	**0.058***
IL-1Ra/IL1	-0.431	**0.008***
IP10/ CXCL10	-0.403	**0.013***
MCP1/ CCL2	-0.249	0.138
MIP-1α /CCL3	-0.243	0.147
MIP-1β/CCL4	-0.154	0.363
PAI1	-0.176	0.298
sCD14	-0.276	0.098
Spearman's Rho Correlation<br>*Statistically significant if p < 0.05		

HIV and CAP group		
	CVF	
Molecules measured in plasma	Correlation coefficient	P value
IL6	-0.247	0.415
IL8	-0.434	0.138
IL10	-0.055	0.859
IL13	-0.362	0.224
IL17A	-0.714	**0.006**
IL-1Ra/IL1	-0.478	0.098
IP10/ CXCL10	-0.022	0.943
MCP1/ CCL2	-0.192	0.529
MIP-1α /CCL3	-0.508	0.076
MIP-1β/CCL4	-0.511	0.074
PAI1	-0.055	0.859
sCD14	-0.033	0.915
Spearman's Rho Correlation<br>*Statistically significant if p < 0.05		

HIV and CAP group		
	CVF	
Molecules measured in plasma	Correlation coefficient	P value
IL1β	-0.534	0.060
IL18	-0.093	0.762
CCL11/eotaxin	-0.050	0.870
VEGF/ VPF	-0.157	0.610
RANTES/CCL5	-0.008	0.980
Pearson's correlation coefficient<br>*Statistically significant if p < 0.05		

HIV group		
	CVF	
Molecules measured in plasma	Correlation coefficient	P value
IL6	-0.219	0.304
IL8	-0.042	0.845
IL10	-0.550	**0.005**
IL13	0.330	0.115
IL17A	0.017	0.939
IL-1Ra/IL1	-0.201	0.347
IP10/ CXCL10	-0.471	**0.020**
MCP1/ CCL2	-0.048	0.824
MIP-1α /CCL3	-0.047	0.829
MIP-1β/CCL4	0.248	0.243
PAI1	-0.189	0.377
sCD14	-0.431	**0.035**
Spearman's Rho Correlation<br>*Statistically significant if p < 0.05		

HIV group		
	CVF	
Molecules measured in plasma	Correlation coefficient	P value
IL1β	0.153	0.475
IL18	-0.132	0.540
CCL11/eotaxin	0.269	0.205
VEGF/ VPF	0.013	0.954
RANTES/CCL5	-0.074	0.730
Pearson's correlation coefficient<br>*Statistically significant if p < 0.05		
